# Cervical Coupling Motion Characteristics in Healthy People Using a Wireless Inertial Measurement Unit

**DOI:** 10.1155/2013/570428

**Published:** 2013-07-09

**Authors:** Hyunho Kim, Sang-Hoon Shin, Jeong-Kyun Kim, Young-Jae Park, Hwan-Sup Oh, Young-Bae Park

**Affiliations:** ^1^Department of Biofunctional Medicine & Diagnostics, College of Korean Medicine, Kyung Hee University, Seoul 130-702, Republic of Korea; ^2^Department of Oriental Biomedical Engineering, College of Health Science, Sangji University, Wonju 220-702, Republic of Korea; ^3^Department of Human Informatics of Korean Medicine, Interdisciplinary Programs, Kyung Hee University, Seoul 130-701, Republic of Korea; ^4^Department of Mechanical Engineering, College of Engineering, Kyung Hee University, Yongin 446-701, Republic of Korea

## Abstract

*Objective*. The objectives were to show the feasibility of a wireless microelectromechanical system inertial measurement unit (MEMS-IMU) to assess the time-domain characteristics of cervical motion that are clinically useful to evaluate cervical spine movement. *Methods*. Cervical spine movements were measured in 18 subjects with wireless IMUs. All rotation data are presented in the Euler angle system. Amount of coupling motions was evaluated by calculating the average angle ratio and the maximum angle ratio of the coupling motion to the primary motion. Reliability is presented with intraclass correlation coefficients (ICC). *Results*. Entire time-domain characteristics of cervical motion were measured with developed MEMS-IMU system. Cervical range of motion (CROM) and coupling motion range were measured with high ICCs. The acquired data and calculated parameters had similar tendency with the previous studies. *Conclusions*. We evaluated cervical motion with economic system using a wireless IMU of high reliability. We could directly measure the three-dimensional cervical motion in degrees in realtime. The characteristics measured by this system may provide a diagnostic basis for structural or functional dysfunction of cervical spine. This system is also useful to demonstrate the effectiveness of any intervention such as conventional medical treatment, and Korean medical treatment, exercise therapy.

## 1. Introduction

Musculoskeletal disorder is one of the most suitable fields which Korean medicine interventions such as acupuncture or moxibustion can be applied to. Although a myriad of studies have been conducted on the efficacy and effectiveness of complementary and alternative medicine to the acute or chronic musculoskeletal disorders [1–5], studies on the evaluation tools for musculoskeletal motion are very rare. Among the various motions, in particular, cervical motion is biomechanically and neurophysiologically complex and depends on various nonskeletal conditions such as muscular, neuromuscular, proprioceptive, and perceptional conditions [6]. By observing cervical range of motion (CROM) and coupling motion, which occurs on the secondary planes relative to the primary plane, we can examine the stability of the cervical function [6–13]. They are also believed to reflect central nervous motor control strategy [14]. CROM and cervical coupling motion have been studied as important elements to diagnose pathological disorders such as clinical instability due to degeneration, disease, or trauma [6, 7, 10, 15, 16]. But, most studies have only measured the maximum coupling motion angles and did not assess the time-domain characteristics, despite their importance.

A motion capture and analysis device is necessary to analyze the time-domain motion characteristic of a subject. These measurements traditionally relied on the naked eye, goniometers, inclinometers, and potentiometers; thus, inaccuracies and limitations have existed [17, 18]. But, with highly developed technology, many studies have been attempted with more accurate devices, such as optoelectronic devices [19, 20], electromagnetic field systems [7], or ultrasonic devices [21, 22]. However, these instruments have some disadvantages. First, they require additional space to fix transmitters and receivers in several strictly calculated spots [20]. Second, these devices require a postmeasurement process to interpolate the data or an error-adjusting system because of disturbance or perturbation of signal by obstacles, and this makes it impossible to acquire real-time data, which is one of the most important disadvantages [23]. Third, these devices are too expensive to be used in clinics or hospitals, as they require several types of transmitters and receivers [24]. Furthermore, the transmitters and receivers can be a physical burden to subjects due to their size and mass.

Many studies have been conducted on body-attached inertial measurement units (IMU) to solve these disadvantages. An IMU is a complex-sensor device consisting of a few accelerometers, gyroscopes, magnetometers, and Kalman filters [25–27]. The IMU allows for an easy analysis of rotation movement because it provides the rotation matrix directly, whereas other instruments should calculate the rotation matrix from the absolute coordinates, so the IMU has been used mainly for animation, game design, or in the gait analysis field. The IMU had initial limitations for measuring minute movements of the human body because of its large size, mass, and wired condition [24, 28–30]. But, size and mass have been reduced dramatically to a few cubic millimeters and a few grams with the development of the microelectromechanical system (MEMS) technology, and it is now possible to measure minute movements without disturbance and to obtain precise human body motion data [31–33]. After the IMU was used for motion analysis, many studies about its validity [34, 35], and reliability [23, 36, 37] in comparison with optoelectronic electromagnetic, and ultrasonic instruments were published. Furthermore, the IMU has become more convenient to use after combining the technology with radio frequency (RF) or Bluetooth wireless systems.

The aim of this study is to show the feasibility of the IMU as a measurement device for analyzing CROM and cervical coupling motion by reporting cervical motion time-domain characteristics in healthy subjects judged by the Neck Disability Index (NDI) questionnaire. We also defined and calculated some parameters to assess cervical motion stability. Previous studies have reported the feasibility of the IMU to measure CROM [38, 39], and other studies have reported coupling motion using the electromagnetic tracking system [14, 40], but few or no study has attempted to measure and analyze cervical motion time-domain characteristics with a wireless IMU.

## 2. Methods

### 2.1. Equipment

Two wireless IMU modules (model EBIMU24G, E2BOX, Seoul, Republic Korea) and one RF receiver (model EBRF24G3CH, E2BOX) were used to measure cervical motion. This IMU module is very small (32 mm × 21 mm × 6.5 mm), and its mass is 7.85 g with a lithium polymer battery attached. The module consists of a 2.4 GHz ISM band wireless transceiver and nine MEMS sensors (three gyroscopes, three accelerometers, and three magnetometers), with nine degrees of freedom. The sensitivities of the sensors are 250–2000 dps (gyroscope), 2–8 g (accelerometer), and 1.3–8.1 gauss (magnetometer). The static accuracy is <0.5°, and dynamic accuracy is <2° according to the 2012 datasheet provided.

### 2.2. NDI Questionnaire

NDI is an adaptation of the Oswestry Low Back Pain questionnaire and consists of ten items, including items for evaluating pain, sleep quality, work ability, driving, and daily living ability. A high score indicates high neck function disability. Test-retest reliability for the NDI is 0.89 [41].

### 2.3. Subjects

Eighteen healthy volunteers were recruited (eight males, eleven females; age, 25–35 years; mean age 27.44 ± 1.89). The subjects were limited to the no- or mild-disability groups as scored by the NDI questionnaire. Subjects were excluded if they had undergone a recent cervical operation or had a disease that could disturb cervical movements. Subjects with 0–4 NDI scores were allocated to the no-disability group, and subjects with 5–14 NDI scores were allocated to the mild-disability group. The mean NDI score of the subjects is 5.65, and the standard deviation is 3.52. The subjects were recruited via advertisements at Kyung Hee University, Oriental Medicine Hospital. This study was approved by the Institutional Review Board of Kyung Hee University, Oriental Medicine Hospital, and all procedures were conducted after informed consent was provided.

### 2.4. Procedure

**Figure 1:**
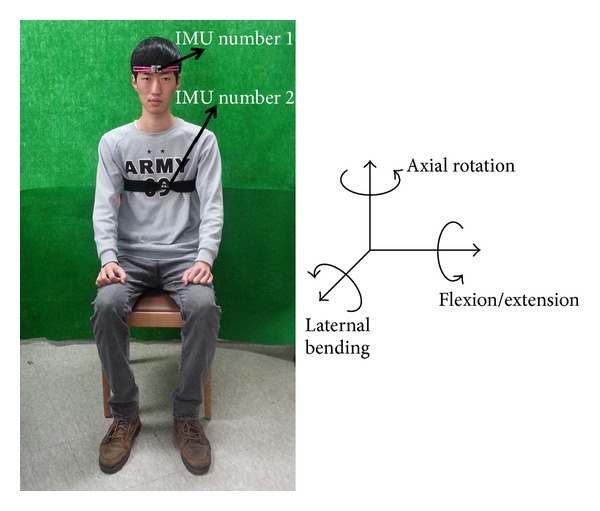
Inertial measurement unit installation and axis presentation.

Subjects were seated on a fixed chair and cervical movement was measured using two wireless MEMS-IMUs at a sampling frequency of 20 Hz. One sensor was placed on the center of the forehead, and the other was placed on the upper one-third point between the suprasternal notch and the xiphoid process. The sensors were fixed with Velcro straps. The sensor located on the forehead measured head motion, and the other sensor was used to compensate for body trunk movement. Device installation is presented in Figure 1.

After instructed to gaze forward naturally, the subjects were instructed to perform three parts of the test. The first part was an exercise. They were instructed how to rotate their heads on three rotation planes such as the transverse plane for left and right axial rotation, the sagittal plane for extension and flexion, and the coronal plane for left and right bending. Subjects were told that all motions should be performed with an almost fixed speed and to rotate or tilt their head as much as possible. The second part was the natural test. Subjects were asked to perform a very natural motion without thinking about adjusting their motion. The last part was the neutral test. Subjects were asked to try not to create a coupling motion, but to move with their head strictly adjusted. All motions were repeated five times. The subjects were asked to close their eyes during all procedures to remove visual stimulation or auto orientation cues. The sequence of tests and movements were fixed, and verbal instructions were recored once and replayed by a computer system for removing the bias induced by instructors. Data were recorded automatically throughout the testing via an RF wireless system. Calibration was performed before each test and measurement.

### 2.5. Data Analysis

Cervical motion data were acquired using Lab VIEW 2010 (National Instruments Inc., Austin, TX, USA) in Euler form for the physical intuitiveness of the Euler angle coordinate system. The sequence of Euler angles is roll-pitch-yaw. All statistical calculations were performed using SPSS Statistics 19 (SPSS, Inc., Chicago, IL, USA).

Data from the sensor attached to the thorax were used to compensate for head movements. We normalized the data along the time axis and calculated the area under curve to estimate the amount of motion. We also calculated intraclass correlation coefficients (ICCs) of CROM and maximum angle of coupling motion by using analysis of variance (ANOVA) to assess reliability of the measurement system.

## 3. Results 

### 3.1. The Cervical Motion Time-Domain Characteristics of One Subject

**Figure 2:**
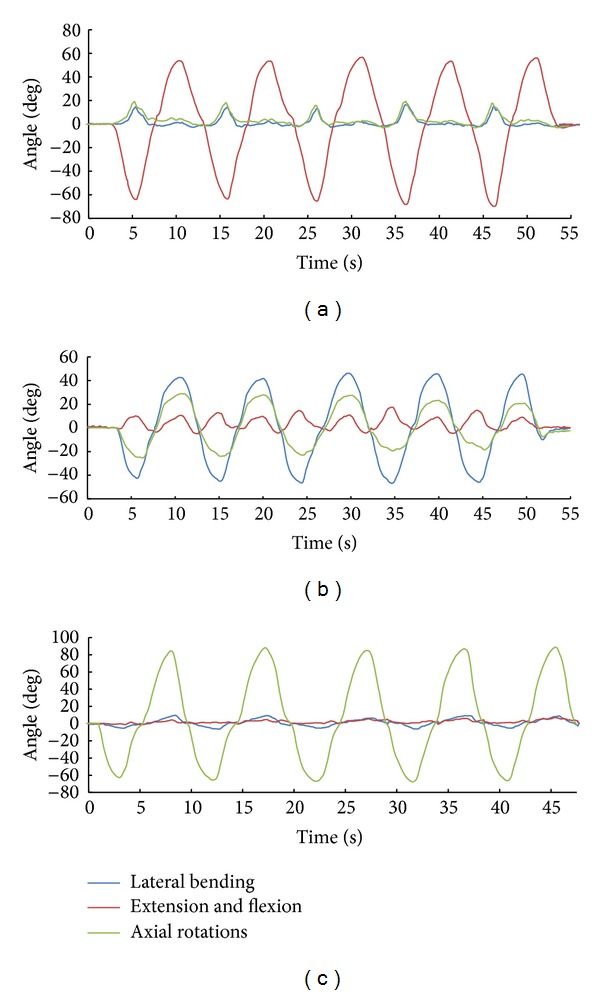
An example of three-dimensional cervical motion patterns described in the Euler rotation coordinates. (a) Measurement when extension and flexion are primary motions. (b) Measurement when lateral bending is primary motions. (c) Measurement when axial rotations are primary motions. Positive values represent flexion, right side bending, and right axial rotation.

Figure 2 shows an example of the cervical motion time-domain characteristics on the natural test. The three curves in the graph are each Euler angle components. The red curve in Figure 2(a) indicates extension and flexion on the sagittal plane, which are primary motions, and the blue and green curves indicate rotation on the coronal and transverse planes, which are the coupling motions in this case. The colored curves in Figures 2(b) and 2(c) represent the same motions as shown in Figure 2(a). Positive values indicate flexion, right lateral bending, and right axial rotation, whereas negative values indicate extension, left lateral bending, and left axial rotation. As shown in Figure 2(a), this subject shows right axial rotation and right lateral bending of about 20 degrees during an extension, whereas almost no rotation or side bending during a flexion. He shows large and complex coupling movement during side bending (Figure 2(b)) but shows little coupling motion of under 10 degrees during axial rotation (Figure 2(c)).

### 3.2. CROM and Maximum Coupling Motion

Similar to traditional instruments, this system can measure the CROM and maximum coupling motion angle.  Table 1 shows the means and standard deviations of the measured CROMs on the natural and neutral tests. We can observe a tendency that the CROMs on the neutral test are less than the CROMs on the natural test. The calculated ICCs of measuring CROM are high (>0.95). The maximum coupling motion angles on the two tests are presented in Table 2 with their ICCs. The average ICC of measuring coupling motion on the natural test is 0.96, and that on the neutral test is 0.94. 

### 3.3. Average Angle Ratio and Maximum Angle Ratio of Coupling Motion

**Figure 3:**
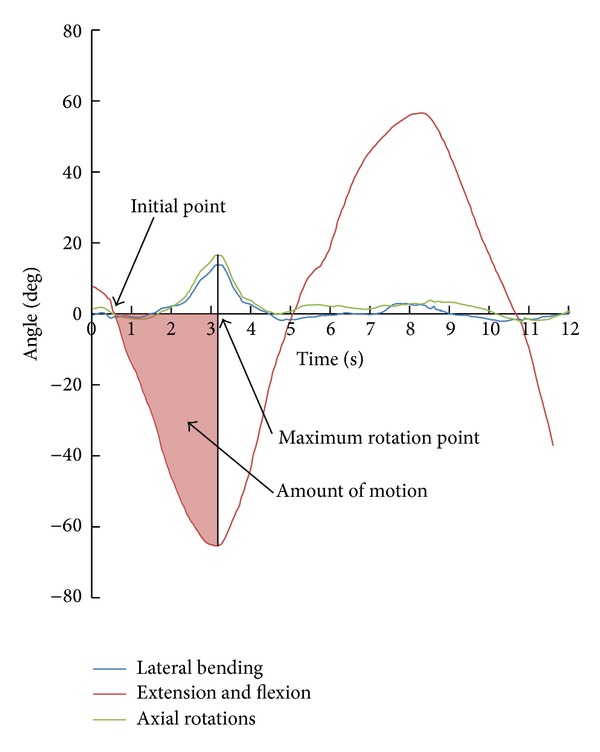
Amount of motion as area under curve. The amount of rotation is defined as the area under curve from the initial point to the maximum rotation point. This area was used to calculate the average angle of coupling motion. Positive values represent flexion, right side bending, and right axial rotation. In this case, there is little coupling motion in flexion.

We set up a parameter, amount of motion, as area under curve. We can calculate the average angle ratio of coupling motion to the primary motion with this amount of motion. This concept is shown in Figure 3. The amount of motion was thought to be proportional to the area under curve from the initial position to the maximum angle because returning from the maximum rotation point is affected by the previously occurred coupling motion. After the area was calculated, it was normalized along the time axis for adjusting the duration difference in each measurement. By normalization along the time axis, we obtained the average coupling motion angle and calculated the average angle ratio of coupling motion to the primary motion. The average angle ratio and maximum angle ratio of coupling motion are shown in Table 3.

## 4. Discussion

### 4.1. Measurements with the Wireless IMU System

We designed and constructed a wireless IMU system to measure cervical motion to overcome the physical, time, and economic disadvantages of prior instruments [20, 23, 24]. The objective of this study was to demonstrate the feasibility of this wireless IMU system to evaluate stability and describe cervical motion. Tables 1 and 2 show the ICCs of this measurement system. The ICCs of the CROM measurements are >0.97 except for one case, and those of the coupling motions are >0.90 except for two cases. This indicates the high reliability of this measurement system and this methodology to measure the cervical motions. ICCs of the coupling motion measurement are generally slightly less than those of the CROM measurements. This could mean that coupling motions cannot be controlled strictly, or a little fluctuation of coupling motion may be occurred in every trial. Nevertheless, the reliability of this system is still high.

Previous studies reported the ICCs of prior instruments. ICC values were >0.791 for measuring CROM and were >0.40 for measuring coupling motions using a three-dimensional electromagnetic motion tracking device [13]. In some cases, almost all ICC values were <0.75 when coupling motion was measured using a three-dimensional electromagnetic motion tracking device [40]. ICCs using the three-dimensional kinematic method were reported >0.74 [42]. A study that measured CROMs with a wired IMU reported ICCs of 0.79–0.99 [38]. Comparing the ICCs of the IMU systems and the prior instruments, the test-retest reliability of the IMU system is relatively very high whether wired or wireless condition. This shows the potential possibility of a wireless IMU system in measuring and evaluating stability or function of the various musculoskeletal joint systems.

### 4.2. Parameters to Analyze the Time-Domain Characteristics

CROM is one of the most well-investigated physical parameter [9] and measuring CROM is important clinically [7, 43]. Additionally, the coupling motion behavioral pattern is also clinically important to diagnose disorders such as degeneration, disease, trauma, or dislocation [6–13]. CROMs in healthy subjects were measured in our study (Table 1), and there is a similar tendency with the previous studies [6, 40]. This result may suggest the possibility of high concurrent validity of this system.

Previous studies reported only maximum values of coupling motion and did not discuss the entire pattern or the time-domain characteristics of coupling motion. Discussion about the entire coupling motion pattern is essential, because the starting point, increasing slope, average coupling angles, and its fluctuation also have clinical information that which muscles are recruited, which nerves are activated, and what pattern the motor control is fulfilled in. The time-domain characteristics of coupling motion can give qualitative and clinical clues about these questions. For example, if coupling motions simultaneously occur with the primary rotation, this case has clinically obvious difference from the case that coupling motions occur after some primary rotation or especially near the maximum point of the primary rotation. But if only maximum angles of coupling motions are reported, the two different cases cannot be distinguished. Likewise, amount of the coupling motion must be considered with amount of the primary motion, because cases of same amount of coupling motion with different maximum range of primary motions must be discussed separately. Cervical movement with more primary ROM in normal range can be understood to have better function or motor control strategy than one with less primary ROM in case that those two have the same amount of coupling motion.

We defined and calculated amount of motion with area under curve to consider the time-domain characteristic including rotation angle and time duration. In addition, to consider the primary motion amount, we defined average angle ratio as the ratio of average coupling motion angle to average primary motion angle. Therefore, one primary motion has two angle ratios according to the two secondary planes (Table 3). Average angle ratios of the coupling motion are large when the primary motion is lateral bending. Maximum angle ratios in primary lateral bending are approximate 30%, and those in primary axial rotations are approximate 10%. These values resemble the results of the previous study that used the prior instruments [10, 44].

### 4.3. Clinical Values of Evaluating the Coupling Motion

Coupling motion generally occurs in healthy subjects, because one motion recruit various muscles. For example, the rectus capitis posterior major, oblique capitis inferior and superior, and splenius muscles are recruited as intrinsic rotators when rotating the head, and the sternocleidomastoid, scalene, upper trapezius, and levator scapular muscles are recruited as extrinsic rotators. Among these, sternocleidomastoid muscle, anterior and middle scalene muscles act as extrinsic flexor muscles. Similarly, the upper trapezius and levator scapular muscles are also extrinsic extensor muscles. So, flexion or extension can occur simultaneously during axial rotation, according to the central nervous system motor control strategies. Coupling motion on the neutral test reflects these inevitably integrated muscle movements because subjects intended to make rotations strictly on only one plane in neutral test. Excessive or distorted coupling motion can occur in asymptotic subjects, who have no clinical symptom such as pain or ROM limitation, because the coupling motion can be a protective postural strategy to avoid pain [14].

We should not directly diagnose muscle or nerve problems only using the amount of coupling motion. Instead, we could observe the entire cervical motion with this measurement system, and by doing so, we could calculate parameters including time-domain information and consider the entire curve for diagnosing diseases or observing progress. A direct correlation between coupling motion pattern and specific musculoskeletal or nervous problem can be estimated if the coupling motion patterns are analyzed by pattern analysis or other data-mining methods, or if imaging studies such as X-ray, computed tomography, or magnetic resonance imaging are combined with this system.

This technique can also be applied to various joint systems, which enables clinicians or physical therapists to evaluate various joint functions, to examine the effect of clinical interventions including complementary and alternative medical treatments, and to preventively care for patients by correcting movement habits. If the proper biofeedback systems are combined with this real-time evaluation, therapeutic approach can be made in the musculoskeletal or neurological rehabilitation fields.

### 4.4. Limitations

We used the NDI index to include only the no- and mild-disability group, but this questionnaire has limitation of being unable to filter out pain-avoidance by creating excessive or limited cervical coupling motion. Although other studies reported an age effect on cervical motion [44, 45], we could not reproduce this effect because the subjects were 25–35 years of age.

## 5. Conclusion

We developed a measurement system using a wireless IMU to evaluate cervical motion quantitatively and qualitatively. Using this system, we were able to measure CROM and coupling motion in real time, so we could observe the entire characteristics of the coupling motions. We calculated the average angle ratios and maximum angle ratio of coupling motions to evaluate the time-domain motion characteristics. These parameters may provide accurate rotation movement data and diagnostic basis for structural or functional dysfunction of the cervical spine with further studies. High test-retest reliability, the resemblance of the results with the previous studies, and the economic advantages of this system may present the potential utility of this system in many aspects.

## Figures and Tables

**Table 1:** Cervical range of motion on the natural test and the neutral test.

Plane	Movement	Natural test (°)		Neutral test (°)	
		Mean ± SD	ICC (95% CI)	Mean ± SD	ICC (95% CI)
Sagittal	Total	116.70 ± 17.26	0.98 (0.96–0.99)	106.76 ± 20.33	0.99 (0.97–1.00)
	Extension	57.23 ± 8.80	0.97 (0.93–0.99)	52.75 ± 9.25	0.95 (0.90–0.98)
	Flexion	58.48 ± 11.83	0.98 (0.96–0.99)	54.02 ± 13.46	0.98 (0.97–0.99)

Coronal	Total	89.42 ± 10.11	0.97 (0.95–0.99)	83.69 ± 13.79	0.99 (0.98–1.00)
	Left	44.15 ± 6.72	0.97 (0.93–0.99)	42.17 ± 7.33	0.98 (0.96–0.99)
	Right	45.28 ± 4.97	0.98 (0.95–0.99)	41.52 ± 7.11	0.98 (0.97–0.99)

Transverse	Total	143.29 ± 17.02	0.99 (0.97–0.99)	134.87 ± 22.43	0.98 (0.97–0.99)
	Left	69.67 ± 10.87	0.99 (0.97–0.99)	64.89 ± 11.74	0.98 (0.96–0.99)
	Right	73.62 ± 10.81	0.99 (0.97–0.99)	69.98 ± 13.99	0.99 (0.98–1.00)

SD: standard deviation; ICC: intraclass correlation coefficient; CI: confidence interval.

**Table 2:** Maximum angle of coupling motions on the natural test and the neutral test.

Primary	Coupling	Natural test (°)		Neutral test (°)	
		Mean ± SD	ICC (95% CI)	Mean ± SD	ICC (95% CI)
Extension	Lateral bending	7.50 ± 4.93	0.95 (0.90–0.98)	5.79 ± 3.60	0.97 (0.93–0.99)
	Axial rotations	8.44 ± 4.56	0.94 (0.88–0.98)	6.52 ± 2.75	0.83 (0.65–0.94)
Flexion	Lateral bending	7.63 ± 5.56	0.98 (0.97–0.99)	5.61 ± 3.08	0.94 (0.87–0.98)
	Axial rotations	7.89 ± 4.31	0.97 (0.94–0.99)	6.18 ± 2.63	0.86 (0.70–0.95)
Left bending	Extension and flexion	13.14 ± 6.22	0.95 (0.90–0.98)	9.93 ± 4.86	0.96 (0.93–0.99)
	Axial rotations	12.21 ± 5.78	0.93 (0.85–0.97)	12.35 ± 5.71	0.95 (0.90–0.98)
Right bending	Extension and flexion	7.72 ± 3.25	0.90 (0.78–0.96)	6.22 ± 2.74	0.93 (0.86–0.97)
	Axial rotations	14.92 ± 6.86	0.97 (0.94–0.99)	14.19 ± 7.34	0.97 (0.95–0.99)
Left rotation	Lateral bending	7.59 ± 4.20	0.97 (0.94–0.99)	5.59 ± 3.12	0.95 (0.91–0.98)
	Extension and flexion	7.38 ± 3.78	0.96 (0.92–0.98)	6.40 ± 3.05	0.94 (0.88–0.98)
Right rotation	Lateral bending	7.09 ± 3.31	0.97 (0.94–0.99)	6.34 ± 3.58	0.96 (0.92–0.99)
	Extension and flexion	7.52 ± 3.32	0.97 (0.93–0.99)	6.22 ± 3.52	0.96 (0.91–0.98)

SD: standard deviation; ICC: intraclass correlation coefficient; CI: confidence interval.

**Table 3:** Average angle ratio and maximum angle ratio of coupling motions on the natural test and the neutral test.

Primary	Coupling	AAR (mean ± SD)		MAR (mean ± SD)	
		Natural test	Neutral test	Natural test	Neutral test
Extension	Lateral bending	0.09 ± 0.05	0.09 ± 0.06	0.13 ± 0.09	0.11 ± 0.07
	Axial rotations	0.14 ± 0.09	0.13 ± 0.07	0.15 ± 0.08	0.13 ± 0.06
Flexion	Lateral bending	0.10 ± 0.06	0.09 ± 0.04	0.12 ± 0.08	0.10 ± 0.05
	Axial rotations	0.12 ± 0.07	0.12 ± 0.09	0.13 ± 0.06	0.12 ± 0.06
Left bending	Extension and flexion	0.27 ± 0.13	0.22 ± 0.13	0.30 ± 0.14	0.24 ± 0.12
	Axial rotations	0.29 ± 0.16	0.16 ± 0.11	0.28 ± 0.12	0.30 ± 0.13
Right bending	Extension and flexion	0.16 ± 0.09	0.29 ± 0.15	0.17 ± 0.07	0.15 ± 0.07
	Axial rotations	0.34 ± 0.18	0.37 ± 0.25	0.34 ± 0.16	0.35 ± 0.19
Left rotation	Extension and flexion	0.09 ± 0.06	0.08 ± 0.04	0.11 ± 0.06	0.08 ± 0.04
	Lateral bending	0.11 ± 0.06	0.12 ± 0.09	0.10 ± 0.05	0.10 ± 0.05
Right rotation	Extension and flexion	0.09 ± 0.05	0.08 ± 0.04	0.09 ± 0.04	0.09 ± 0.04
	Lateral bending	0.13 ± 0.08	0.12 ± 0.09	0.10 ± 0.05	0.09 ± 0.06

SD: standard deviation; AAR: average angle ratio; MAR: Maximum angle ratio.

## References

[1] Lee H, Lee JH, Choi TY, Lee MS, Lee H, Shin BC (2013). Acupuncture for acute low back pain: a systematic review. *Clinical Journal of Pain*.

[2] Xu M, Yan S, Yin X (2013). Acupuncture for chronic low back pain in long-term follow-up: a meta-analysis of 13 randomized controlled trials. *American Journal of Chinese Medicine*.

[3] Kwak H, Kim J, Park J (2012). Acupuncture for Whiplash-associated disorder: a randomized, waiting-list controlled, pilot trial. *European Journal of Integrative Medicine*.

[4] Tobbackx Y, Meeus M, Wauters L (2013). Does acupuncture activate endogenous analgesia in chronic whiplash-associated disorders? A randomized crossover trial. *European Journal of Pain*.

[5] Hutchinson AJP, Ball S, Andrews JCH, Jones GG (2012). The effectiveness of acupuncture in treating chronic non-specific low back pain: a systematic review of the literature. *Journal of Orthopaedic Surgery and Research*.

[6] Malmström E, Karlberg M, Fransson PA, Melander A, Magnusson M (2006). Primary and coupled cervical movements: the effect of age, gender, and body mass index. A 3-dimensional movement analysis of a population without symptoms of neck disorders. *Spine*.

[7] Koerhuis CL, Winters JC, van der Helm FCT, Hof AL (2003). Neck mobility measurement by means of the “Flock of Birds” electromagnetic tracking system. *Clinical Biomechanics*.

[8] Hermann KM, Reese CS, Jette AM (2001). Relationships among selected measures of impairment, functional limitation, and disability in patients with cervical spine disorders. *Physical Therapy*.

[9] Strimpakos N, Sakellari V, Gioftsos G (2005). Cervical spine ROM measurements: optimizing the testing protocol by using a 3D ultrasound-based motion analysis system. *Cephalalgia*.

[10] Feipel V, Rondelet B, Le Pallec J, Rooze M (1999). Normal global motion of the cervical spine: an electrogoniometric study. *Clinical Biomechanics*.

[11] Lee S, Draper ERC, Hughes SPF (1997). Instantaneous center of rotation and instability of the cervical spine: a clinical study. *Spine*.

[12] Cook C, Hegedus E, Showalter C, Sizer PS (2006). Coupling behavior of the cervical spine: a systematic review of the literature. *Journal of Manipulative and Physiological Therapeutics*.

[13] Guo L, Lee S, Lin C (2012). Three-dimensional characteristics of neck movements in subjects with mechanical neck disorder. *Journal of Back and Musculoskeletal Rehabilitation*.

[14] Woodhouse A, Vasseljen O (2008). Altered motor control patterns in whiplash and chronic neck pain. *BMC Musculoskeletal Disorders*.

[15] Pearcy MJ, Tibrewal SB (1984). Axial rotation and lateral bending in the normal lumbar spine measured by three-dimensional radiography. *Spine*.

[16] Panjabi MM, Oda T, Crisco JJ, Dvorak J, Grob D (1993). Posture affects motion coupling patterns of the upper cervical spine. *Journal of Orthopaedic Research*.

[17] Buck CA, Dameron FB, Dow MJ, Skowlund HV (1959). Study of normal range of motion in the neck utilizing a bubble goniometer. *Archives of Physical Medicine and Rehabilitation*.

[18] Capuano-Pucci D, Rheault W, Aukai J, Bracke M, Day R, Pastrick M (1991). Intratester and intertester reliability of the cervical range of motion device. *Archives of Physical Medicine and Rehabilitation*.

[19] Canseco K, Albert C, Long J, Khazzam M, Marks R, Harris GF (2011). Postoperative foot and ankle kinematics in rheumatoid arthritis. *Journal of Experimental and Clinical Medicine*.

[20] Rahni AAA, Yahya I Obtaining translation from a 6-DOF MEMS IMU—an overview.

[21] Castro WHM, Sautmann A, Schilgen M, Sautmann M (2000). Noninvasive three-dimensional analysis of cervical spine motion in normal subjects in relation to age and sex: an experimental examination. *Spine*.

[22] Park K, Cynn H, Kwon O (2011). Effects of the abdominal drawing-in maneuver on muscle activity, pelvic motions, and knee flexion during active prone knee flexion in patients with lumbar extension rotation syndrome. *Archives of Physical Medicine and Rehabilitation*.

[23] Chung PYM, Ng GYF (2011). Comparison between an accelerometer and a three-dimensional motion analysis system for the detection of movement. *Physiotherapy*.

[24] Liu T, Inoue Y, Shibata K, Tang X A wearable inertial sensor system for human motion analysis.

[25] Sessa S, Zecca M, Lin Z Ultra-miniaturized WB-3 Inertial Measurement Unit: performance evaluation of the attitude estimation.

[26] Lin Z, Zecca M, Sessa S Development of an ultra-miniaturized inertial measurement unit WB-3 for human body motion tracking.

[27] Sabatini AM, Martelloni C, Scapellato S, Cavallo F (2005). Assessment of walking features from foot inertial sensing. *IEEE Transactions on Biomedical Engineering*.

[28] Godfrey A, Conway R, Meagher D, ÓLaighin G (2008). Direct measurement of human movement by accelerometry. *Medical Engineering and Physics*.

[29] Ochi F, Abe K, Ishigami S, Otsu K, Tomita H Trunk motion analysis in walking using gyro sensors.

[30] Tao L, Inoue Y, Shibata K, Morioka H Development of wearable sensor combinations for human lower extremity motion analysis.

[31] Saber-Sheikh K, Bryant EC, Glazzard C, Hamel A, Lee RYW (2010). Feasibility of using inertial sensors to assess human movement. *Manual Therapy*.

[32] Cardarelli D An integrated MEMS inertial measurement unit.

[33] Warnasch A, Killen A Low cost, high G, Micro Electro-Mechanical Systems (MEMS), Inertial Measurements Unit (IMU) program.

[34] Ito T Walking motion analysis using 3D acceleration sensors.

[35] Kavanagh JJ, Menz HB (2008). Accelerometry: a technique for quantifying movement patterns during walking. *Gait and Posture*.

[36] Moe-Nilssen R (1998). Test-retest reliability of trunk accelerometry during standing and walking. *Archives of Physical Medicine and Rehabilitation*.

[37] Henriksen M, Lund H, Moe-Nilssen R, Bliddal H, Danneskiod-Samsøe B (2004). Test-retest reliability of trunk accelerometric gait analysis. *Gait and Posture*.

[38] Theobald PS, Jones MD, Williams JM (2012). Do inertial sensors represent a viable method to reliably measure cervical spine range of motion?. *Manual Therapy*.

[39] Jasiewicz JM, Treleaven J, Condie P, Jull G (2007). Wireless orientation sensors: their suitability to measure head movement for neck pain assessment. *Manual Therapy*.

[40] Guo L, Yang C, Yang C, Hou Y, Chang J, Wu W (2011). The feasibility of using electromagnetic motion capture system to measure primary and coupled movements of cervical spine. *Journal of Medical and Biological Engineering*.

[41] Vernon H, Mior S (1991). The neck disability index: a study of reliability and validity. *Journal of Manipulative and Physiological Therapeutics*.

[42] Bulgheroni MV, Antonaci F, Ghirmai S, Sandrini G, Nappi G, Pedotti A (1998). A 3D kinematic method for evaluating voluntary movements of the cervical spine in humans. *Functional Neurology*.

[43] Lee H, Nicholson LL, Adams RD (2004). Cervical range of motion associations with subclinical neck pain. *Spine*.

[44] Sforza C, Grassi G, Fragnito N, Turci M, Ferrario VF (2002). Three-dimensional analysis of active head and cervical spine range of motion: effect of age in healthy male subjects. *Clinical Biomechanics*.

[45] Youdas JW, Garrett TR, Suman VJ, Bogard CL, Hallman HO, Carey JR (1992). Normal range of motion of the cervical spine: an initial goniometric study. *Physical Therapy*.

